# Functional Characterization of Enzymatic Steps Involved in Pyruvylation of Bacterial Secondary Cell Wall Polymer Fragments

**DOI:** 10.3389/fmicb.2018.01356

**Published:** 2018-06-27

**Authors:** Fiona F. Hager, Arturo López-Guzmán, Simon Krauter, Markus Blaukopf, Mathias Polter, Inka Brockhausen, Paul Kosma, Christina Schäffer

**Affiliations:** ^1^NanoGlycobiology Unit, Department of NanoBiotechnology, Universität für Bodenkultur Wien, Vienna, Austria; ^2^Division of Organic Chemistry, Department of Chemistry, Universität für Bodenkultur Wien, Vienna, Austria; ^3^Department of Biomedical and Molecular Sciences, Queen’s University, Kingston, ON, Canada

**Keywords:** secondary cell wall polymer, SLH domain, glycosyltransferase, pyruvyltransferase, multi-enzyme assay

## Abstract

Various mechanisms of protein cell surface display have evolved during bacterial evolution. Several Gram-positive bacteria employ S-layer homology (SLH) domain-mediated sorting of cell-surface proteins and concomitantly engage a pyruvylated secondary cell-wall polymer as a cell-wall ligand. Specifically, pyruvate ketal linked to β-D-ManNAc is regarded as an indispensable epitope in this cell-surface display mechanism. That secondary cell wall polymer (SCWP) pyruvylation and SLH domain-containing proteins are functionally coupled is supported by the presence of an ortholog of the predicted pyruvyltransferase CsaB in bacterial genomes, such as those of *Bacillus anthracis* and *Paenibacillus alvei*. The *P. alvei* SCWP, consisting of pyruvylated disaccharide repeats [→4)-β-D-GlcNAc-(1→3)-4,6-Pyr-β-D-ManNAc-(1→] serves as a model to investigate the widely unexplored pyruvylation reaction. Here, we reconstituted the underlying enzymatic pathway *in vitro* in combination with synthesized compounds, used mass spectrometry, and nuclear magnetic resonance spectroscopy for product characterization, and found that CsaB-catalyzed pyruvylation of β-D-ManNAc occurs at the stage of the lipid-linked repeat. We produced the *P. alvei* TagA (PAV_RS07420) and CsaB (PAV_RS07425) enzymes as recombinant, tagged proteins, and using a synthetic 11-phenoxyundecyl-diphosphoryl-α-GlcNAc acceptor, we uncovered that TagA is an inverting UDP-α-D-ManNAc:GlcNAc-lipid carrier transferase, and that CsaB is a pyruvyltransferase, with synthetic UDP-α-D-ManNAc and phosphoenolpyruvate serving as donor substrates. Next, to substitute for the UDP-α-D-ManNAc substrate, the recombinant UDP-GlcNAc-2-epimerase MnaA (PAV_RS07610) of *P. alvei* was included in this *in vitro* reconstitution system. When all three enzymes, their substrates and the lipid-linked GlcNAc primer were combined in a one-pot reaction, a lipid-linked SCWP repeat precursor analog was obtained. This work highlights the biochemical basis of SCWP biosynthesis and bacterial pyruvyl transfer.

## Introduction

The cell surface influences the physicochemical properties of a bacterium, its physiology, life-style, and fitness in a competitive habitat. While the cell surface of any bacterium is composition- and structure-wise unique and, thus, can be regarded as a “bacterial bar code," distinct mechanisms for the robust and, frequently, multivalent display of proteins have evolved. Cell surface display mechanisms are usually based on the fusion of a cell wall targeting motif to the proteins deemed for display, working in concert with a cell wall ligand (Lee et al., 2003; Desvaux et al., 2006).

Several Gram-positive bacteria employ SLH domain-mediated sorting of cell surface proteins and concomitantly engage a pyruvylated SCWP (also known as cell wall glycopolymer) as a cell wall ligand. Specifically, pyruvate ketal linked to β-D-ManNAc is regarded an indispensable and ancestral epitope in this cell surface display mechanism (Mesnage et al., 2000; Cava et al., 2004; Kern et al., 2010). There are over 54,000 specific hits within the conserved protein domain family SLH (pfam00395) (Marchler-Bauer et al., 2015), widely in the phyla firmicutes, cyanobacteria, and actinobacteria, among others, underlining the prevalence of this protein domain in bacteria. The functional coupling of SLH domain containing proteins (SLH proteins) and SCWP pyruvylation is substantiated by the finding that several bacteria – including, e.g., *Bacillus anthracis* (Forsberg et al., 2012), *Bacillus cereus* strains (Forsberg et al., 2011), *Lysinibacillus sphaericus* (Ilk et al., 1999), *Thermoanaerobacterium thermosulfurigenes* (May et al., 2006), and *Paenibacillus alvei* (Schäffer et al., 2000), all synthesize a suite of SLH proteins, contain pyruvate in their cell wall and have an ortholog of the CsaB enzyme predicted to catalyze the transfer of pyruvate ketal to β-D-ManNAc (Mesnage et al., 2000). Experimental data on CsaB is available for *B. anthracis* (Mesnage et al., 2000; Kern et al., 2010) and *Thermus thermophilus* (Cava et al., 2004), where *csaB* deficient mutants revealed a drastically reduced pyruvic acid content (by ∼98%) in comparison to the parent strain supporting pyruvyl transfer activity of CsaB.

Considering the predictably wide-spread occurrence of this protein cell surface display mechanism – in both pathogenic and non-pathogenic bacteria - it is surprising how little is known about the biosynthesis of pyruvylated SCWPs and the involved enzymes. Pyruvylated SCWPs are covalently linked to muramic acid residues of the peptidoglycan backbone and differ structurally from the well investigated teichoic/teichuronic acids (Archibald et al., 1993; Mesnage et al., 2000; Messner et al., 2009; Schade and Weidenmaier, 2016; Rajagopal and Walker, 2017), with the lack of repetitive alditol phosphates and phosphodiester bonds as the most evident differences (Sára, 2001; Schäffer and Messner, 2005).

The SCWP of *P. alvei* CCM 2051^T^ (referred to as *P. alvei* throughout the manuscript) - the model organism of the current study - is composed of, on average, 11 pyruvylated disaccharide repeats with the structure [→4)-β-D-GlcNAc-(1→3)-4,6-Pyr-β-D-ManNAc-(1→] (Schäffer et al., 2000). This SCWP is pivotal to the integrity of the bacterium’s cell wall due to its interaction with *P. alvei*’s abundant S-layer protein SpaA (Janesch et al., 2013b) and the cell surface protein SlhA (Janesch et al., 2013a), both possessing three dedicated SLH domains, each, involved in SCWP binding interactions (Ryan J. Blackler, Arturo López-Guzmán, Fiona F. Hager, Gudrun Martinz, Susannah M. L. Gagnon, Omid Haji-Ghassemi, Bettina Janesch, Paul Messner, Paul Kosma, Christina Schäffer and Stephen V. Evans, unpublished data). In close vicinity to the *P. alvei spaA* and *slhA* genes on the bacterial genome, five ORFs – named, based on a pBLAST search in relation to the sequenced genome of *P. alvei* DSM 29, *orf1* (PAV_RS07430), *csaB* (PAV_RS07425), *tagA* (PAV_RS07420), *tagO* (PAV_RS07415), and *orf7* (PAV_RS07395) - were identified, which we predicted to constitute an SCWP biosynthesis gene locus (Zarschler et al., 2010). While *P. alvei* does not harbor teichoic acids in its cell wall, TagA and TagO from that gene locus show amino acid sequence similarity to the TagA and TagO enzymes from the polyribitol wall teichoic acid biosynthesis pathway in *Staphylococcus aureus* (Tag A, 36.9% and Tag O, 42.0%) and *Bacillus subtilis* (Tag A, 37.2% and Tag O, 32.0%) (Ginsberg et al., 2006; Brown et al., 2010, 2013). The first enzyme in this pathway, TagO, is an integral membrane protein that transfers GlcNAc-phosphate from UDP-GlcNAc to an undecaprenylphosphate carrier lipid embedded in the cytoplasmic membrane (Weidenmaier et al., 2004; D’Elia et al., 2006b). The lipid-linked monosaccharide is then elongated to a disaccharide by the UDP-ManNAc transferase TagA (Zhang et al., 2006; Brown et al., 2008; D’Elia et al., 2009). While this lipid-linked disaccharide constitutes the platform for the subsequent steps of wall teichoic acid biosynthesis (Lazarevic et al., 2002; Swoboda et al., 2010; Kawai et al., 2011; Zilla et al., 2015; Schaefer et al., 2017), it is equivalent to the disaccharide substrate needed to generate the repeat backbone of the *P. alvei* SCWP (Schäffer et al., 2000).

Information on the pyruvylation step in SCWP biosynthesis, or pyruvylation of sugars in general, is scarce. Pyruvyl transfer activity of CsaB in the context of bacterial cell wall synthesis can be indirectly inferred from data on a few bacteria, in which *csaB* deletion mutants could be created by using sophisticated strategies. A *B. anthracis* Δ*csaB* mutant, for instance, showed S-layer deficiency – probably due to the loss of the pyruvylated motif in the SCWP cell wall ligand – and atypical cell morphology forming long chains of incompletely separated cells (Mesnage et al., 2000; Kern et al., 2010). However, while the structure of the *B. anthracis* SCWP is known (Forsberg et al., 2012), loss of SCWP pyruvylation in a *B. anthracis* mutant has not been demonstrated experimentally. Further, Wang et al. (2013) described *csaB* mutant cells of *B. cereus* G9241, which showed reduced pathogenicity in causing anthrax-like disease in mice. In contrast, in *P. alvei*, attempts to delete *csaB* have not been successful (Bettina Janesch, Fiona F. Hager, Christina Schäffer, unpublished data), potentially indicating essentiality of the enzyme.

Recent data comes from the pyruvyltransferase Pvg1p of the fission yeast *Schizosaccharomyces pombe*, which carries pyruvic acid 4,6-ketal-linked to galactose (PvGal) as decoration of its *N*-glycans (Gemmill and Trimble, 1996). The recombinant Pvg1p enzyme transfered pyruvyl residues from PEP specifically to β-linked galactose as present in *p*-nitrophenyl-β-Gal (pNP-β-Gal) or pNP-β-lactose (Yoritsune et al., 2013). The crystal structure of the pyruvyltransferase Pvg1p was obtained at a resolution of 2.46 Å, which served as a basis for enzyme/substrate modeling (Higuchi et al., 2016). PvGal biosynthesis was also studied *in vitro* in the context of the therapeutic potential of the *Bacteroides fragilis* capsular polysaccharide A (Sharma et al., 2017).

The current study focuses on the predicted UDP-ManNAc transferase TagA and the predicted pyruvyltransferase CsaB from the *P. alvei* SCWP biosynthesis gene locus. We produced these enzymes, and also the bacterium’s cognate UDP-GlcNAc-2-epimerase MnaA, in *Escherichia coli* and used the recombinant, purified proteins in *in vitro* enzyme assays, together with a chemically synthesized GlcNAc-PP-UndPh acceptor (Wang et al., 2014) and PEP as donor substrates, and, in some assays, UDP-ManNAc. In a one-pot reaction, a nature-analogous, lipid-linked SCWP repeat could be obtained, as was confirmed by mass spectrometry and NMR spectroscopy. It was found that pyruvylation of the β-D-ManNAc residue occurs at the lipid-linked disaccharide stage. This study contributes to our understanding of how SCWPs other than teichoic acids/teichuronic acids are biosynthesized and specifically sheds light on the pyruvylation step. Pyruvylation might constitute a valuable target for interfering with the bacterial cell wall composition and, thereby, also bacterial pathogenicity.

## Materials and Methods

### Bacterial Strains and Culture Conditions

*Paenibacillus alvei* CCM 2051^T^ (wild-type strain; Czech Collection of Microorganisms, CCM; Brno, Czech Republic) was grown at 37°C and 160 rpm in Luria-Bertani (LB) broth or on LB agar plates. *E. coli* DH5α and BL21 (DE3) (Invitrogen) were cultivated in selective LB medium (agar or broth) supplemented with 100 μg/ml ampicillin (Amp) at 37°C with 180 rpm.

### RNA Purification and Reverse Transcription-PCR

Total RNA was extracted from *P. alvei* using the PureLink RNA Mini Kit (Thermo Fisher Scientific) and subsequently on-column purified with Purelink DNAse (Thermo Fisher Scientific) and TurboDNAse (Ambion) to remove DNA contamination. cDNA was then generated using the MultiScribe Reverse Transcriptase from the High-Capacity cDNA Reverse Transcription Kit (Thermo Fisher Scientific) using 500 ng of total RNA. One-twentieth of the cDNA reaction mixture was used as template for PCR using the Phusion High-Fidelity DNA polymerase (Thermo Fisher Scientific). The produced cDNA was amplified by PCR using primer pairs csaB_for/tagA_rev and tagA_for/ tagO_rev spanning, from 5′ to 3′, the genes *csaB* and *tagA*, and *tagA* and *tagO*, respectively. Further, the region spanning *csaB* and *tagO* was bridged using csaB_for/tagO_rev. As a positive control, gDNA was used, whereas DNase I-treated RNA without the cDNA-generating step as well as NRT (reverse transcription reaction missing reverse transcriptase) served as a control for contamination of total RNA with chromosomal DNA. PCR reaction products were investigated by agarose gel electrophoresis. Primers for RT-PCR were purchased from ThermoFisher Scientific and are listed in **Table 1**.

**Table 1:** Oligonucleotide primers used for PCR amplification reactions.

Gene	Primer	Sequence (5′–3′)
*csaB*	csaB_for	GGCGCAAGTGATGAAGAAGC
*tagA*	tagA_rev	GAAGCTGCCCCCGACACCCA
*tagA*	tagA_for	GCTGTTCGTTGCTCGTGGAG
*tagO*	tagO_rev	CCAGAGTCCCCCATAAAGAT
*mnaA*-His_6_	MnaA_fwd_*Nde*I	GCGCGGGCATATGTCCAAAGTGAAAGTA
*mnaA*-His_6_	MnaA_rev_*Xho*I	GCGCGGGCTCGAGATTTGTGAACTTTGT
MBP-*tagA*	TagA_fwd_*Eco*RI	CGATGGAATTCATGTTAGAAATGAAGGAT
MBP-*tagA*	TagA_rev_*Pst*I	CGATGCTGCAGTTACTGCACTTTTGTGGG
*csaB*-His_6_	CsaB_fwd_*Nde*I	CTGTGGCATATGGCGTCCAAAGCTAC AAGAATAGTACTTTCCGGATATTACGGATTC
*csaB*-His_6_	CsaB_rev_*Xho*I	GACCTACTCGAGCGCCTTATGACGCAGCCAC TTCACAATTTGTTGCGCTGGCTGTTCTGCTT

### DNA Techniques and Plasmid Construction

Genomic DNA of *P. alvei* was isolated from 5 ml of bacterial culture (Cheng and Jiang, 2006). For the purification of DNA fragments and digested plasmids from agarose gels, the GeneJET^TM^ Gel Extraction Kit (Fermentas) was used. Isolation of plasmid DNA from transformed *E. coli* cells was done using the GeneJET^TM^ Plasmid Miniprep Kit (Fermentas). Primers for PCR and DNA sequencing were purchased from ThermoFisher Scientific and are listed in **Table 1**. For the amplification of genes from gDNA of *P. alvei*, the Phusion High-Fidelity DNA Polymerase (Fermentas) and the thermal cycler My CyclerTM (Bio-Rad) were used.

For recombinant protein expression in *E. coli* BL21 (DE3) cells, plasmids encoding MnaA and CsaB as C-terminal His_6_-tag fusion constructs were created by PCR using *P. alvei* gDNA as a template and the primer pairs MnaA_fwd_*Nde*I/ MnaA_rev_*Xho*I and CsaB_fwd_*Nde*I/CsaB_rev_*Xho*I (**Table 1**), respectively, to amplify the 1175-bp *mnaA* (PAV_RS07420) and the 1209-bp *csaB* (PAV_RS07425) genes. The amplification products were digested with *Nde*I/*Xho*I and cloned into *Nde*I/*Xho*I-linearized pET22b(+) (Novagen) for expression of His_6_-tagged proteins. Recombinant TagA was produced as a fusion protein with the maltose binding protein (MBP) located at its N-terminus, because of a 10-fold higher protein expression rate in comparison to His_6_-tagged TagA (Fiona F. Hager, Christina Schäffer, unpublished observation). The primer pair TagA_fwd_*Eco*RI/TagA_rev_*Pst*I was used to produce the 781-bp *tagA* (PAV_RS07420) amplicon, which was digested with *Eco*RI/*Pst*I and cloned into *Eco*RI/*Pst*I-linearized pMAL_c2E vector (NEB). All three constructs were chemically transformed into *E. coli* DH5α cells for the amplification of plasmid DNA. Transformants were screened by colony PCR using RedTaq ReadyMix PCR mix (Sigma-Aldrich) and confirmed by restriction mapping and sequencing (Microsynth).

### Heterologous Overexpression and Purification of Recombinant Enzymes

The plasmids encoding *P. alvei* MnaA-His_6_, CsaB-His_6_, and MBP-TagA, and named pET22b_mnaA, pET22b_csaB, and pMAL_tagA, respectively, were heat-shock-transformed into *E. coli* BL21 (DE3) cells. Selected transformed cells were grown in LB medium to the mid-exponential growth phase (OD_600_ 0.5–0.8), and protein expression was induced with a final concentration of 0.6 mM isopropyl-β-D-thiogalactopyranosid (IPTG). After induction, the bacterial cultures were grown for 4 h at 37°C and 180 rpm followed by harvest through centrifugation (5,500 ×*g*, 20 min, 8°C).

For nickel affinity chromatography of MnaA-His_6_ and CsaB-His_6_, the cell pellets of 500 ml bacterial culture containing the C-terminally His_6_-tagged enzymes were resuspended in lysis buffer (50 mM sodium phosphate, pH 7.5, 200 mM NaCl, supplemented with 10 mM imidazole and EDTA-free protease inhibitor mixture; cOmplete, Roche Applied Science). Cells were disrupted by sonication applying nine pulses of 20 s (Branson Sonifier 250; output 8, duty cycle 45%, 10 s breaks), each, and lysates were ultra-centrifuged (80,695 ×*g*, 20 min, 4°C) to remove cell debris. The supernatant fraction (cell crude extract) was incubated for 30 min with 2 ml of nickel–nitrilotriacetic acid resin in a column (Qiagen) equilibrated in lysis buffer using a flow rate of 1.0 ml/min. After recovery of the flow through, the column was washed with lysis buffer containing increasing imidazole concentrations of 20 and 50 mM, 10 column volumes, each. Finally, the protein of interest was eluted with 5 ml of 250 mM imidazole in lysis buffer. Fractions containing MnaA-His_6_ and CsaB-His_6_, respectively, as determined by 10% SDS-PAGE (Laemmli, 1970) after Coomassie Brilliant Blue G250 staining, were pooled and extensively dialyzed against 25 mM sodium phosphate buffer, pH 7.5, at RT, to remove imidazole.

The MBP-TagA fusion protein was purified over an amylose resin (NEB; 5 ml of resin per liter of bacterial culture) according to the manufacturer’s protocol. Protein elution was done with 20 mM Tris/HCl, pH 7.5, containing 200 mM NaCl, 1 mM EDTA, and 10 mM maltose. Fractions containing the protein of interest according to Coomassie Brilliant Blue G250-stained 10% SDS-PAGE were pooled and extensively dialyzed at RT against 25 mM bis(2-hydroxyethyl)amino-Tris(hydroxymethyl)methane (bis-Tris-propane), pH 7.8, for the TagA *in vitro* activity assay, and against 25 mM sodium phosphate buffer, pH 7.5, for MnaA/TagA co-incubation, to remove maltose.

### Protein Analytical Methods

The recombinant proteins were verified by Western-blotting using in the case of MnaA-His_6_ and CsaB-His_6_ an anti-His_6_ antibody (Sigma-Aldrich) and for MBP-TagA, an anti-MBP antibody (Thermo Scientific). The protein concentration of the recombinant, tagged enzymes was measured spectrophotometrically and calculated using the A_280_ extinction coefficient and molecular weight obtained from the exPASy ProtParam tool^1^ and by the Bradford protein assay (Bradford, 1976).

### Chemical Synthesis of UDP-α-D-ManNAc

UDP-α-D-ManNAc was chemically synthesized as a donor substrate for the TagA reaction, essentially following established protocols (Yamazaki et al., 1980; Freese and Vann, 1996; Ginsberg et al., 2006) (Supplementary Scheme S1). Starting from 2-acetamido-2-deoxy-D-mannopyranose (compound **6**), the triethylammonium salt compound **7** (triethylammonium 2-acetamido-2-deoxy-α-D-mannopyranosyl phosphate) was synthesized. Compound **7** (46 mg, 0.092 mmol) was reacted with uridine 5′-monophosphomorpholidate (153 mg, 0.223 mmol) in 8 ml of freshly distilled dried pyridine under argon atmosphere at RT for 7 days and then concentrated *in vacuo* at 30°C. The product was purified by using a Bio-Scale Mini Macro-Prep High Q anion exchange cartridge (Bio-Rad) with a gradient (0–100%) of 0.25 M triethylammonium bicarbonate buffer, pH 8.0, and lyophilized to give compound **8** [triethylammonium uridine 5′-(2-acetamido-2-deoxy-α-D-mannopyranosyl diphosphate)].

### Enzymatic Synthesis of UDP-α-D-ManNAc Using *P. alvei* UDP-GlcNAc Epimerase MnaA

The enzymatic assay of MnaA was set up based on a published protocol (Blume, 2003), with several modifications. Briefly, purified *P. alvei* MnaA-His_6_ (45 μg) was incubated with UDP-GlcNAc (Sigma-Aldrich) at a concentration of 1.3 or 0.5 mM, in 46 mM sodium phosphate buffer, pH 7.5, supplemented with 11 mM MgCl_2_, in a total reaction volume of 192.5 μl at 37°C for up to 30 min; individual reactions were done and stopped in intervals of 5 min. After heat-inactivation (100°C, 1 min), the mixture was centrifuged (10,000 × *g*, 5 min, RT), and the supernatant was analyzed by reversed-phase (RP) HPLC (Thermo Scientific/Dionex; Ultimate 3000 Standard LC System) on a Hyperclone 5 μ ODS column (Phenomenex, 150 mm × 4.6 mm, 5 μ) using 0.4 M sodium phosphate buffer, pH 6.1, with a flow rate of 0.6 ml/min as eluent (Zolghadr et al., 2015). Peaks were identified using UDP (1 nmol), UDP-GlcNAc (5 nmol), and UDP-ManNAc (5 nmol) as standards; detection was done at 254 nm. Epimerization rates by MnaA were calculated from the integrated peak areas using software provided by the Ultimate 3000 Standard LC System.

### Sep-Pak Purification of Enzyme Products

Purification of products from *in vitro* enzyme reactions (as described below) was done using a (C18) Sep-Pak classic cartridge (Waters; 360 mg sorbens). The cartridge was equilibrated with 3 ml of methanol followed by 6 ml of H_2_O immediately prior to application of the reaction mixture. The samples were loaded on the cartridge by gravity flow, and the flow-through was collected. Subsequently, the cartridge was eluted with 4 ml of H_2_O (1-ml fractions) for removal of excess of UDP-ManNAc and hydrophilic components (bis-Tris-propane, sodium phosphate, MgCl_2_, or potential enzyme products that are more hydrophilic than the initial acceptor substrate), followed by 3 ml of methanol (1.5 ml fractions); fractions were collected and dried *in vacuo* using a SpeedVac centrifuge (Thermo Fisher Scientific).

### LC-ESI-MS of Purified Enzyme Products

Fractions obtained after (C18) Sep-Pak purification were dissolved in a 1:1-solution of H_2_O and acetonitrile, and 10 μl of the sample solutions were analyzed by LC (C4)-ESI-MS using a Phenomenex Jupiter 5 μ C4, 300 Å, 150 mm × 2 mm column coupled to an LC-MS device (Shimadzu LC 10 system, Shimadzu 2020 mass spectrometer, Alltech ELSD 3300). The elution profile used during analysis was a gradient of 5–100% CH_3_CN (0–2 min: 5% CH_3_CN; 2–10 min: 5–100%, 15–16 min: 100–5%, 16–22 min: 5%) at a flow of 0.5 ml/min and a column temperature of 40°C. The eluate was directed into the ESI source for mass detection in the range of 50–2000 with a scan speed of 2143 μ/s, and the masses were evaluated using the integrated software, Shimadzu – LabSolutions V. 5.42 SP4.

### NMR Spectroscopy of Enzyme Products

NMR spectra of enzyme products were recorded at 297 K in 99.9% D_2_O (0.4 ml) in a Shigemi tube with a Bruker Avance III 600 spectrometer (^1^H at 600.13 MHz, ^13^C at 150.9 MHz, ^31^P at MHz at 242.9 MHz), using standard Bruker NMR software. ^1^H NMR spectra were referenced to 2,2-dimethyl-2-silapentane-5-sulfonic acid (δ 0.0), ^13^C NMR spectra were referenced to external dioxane (δ 67.40), and ^31^P spectra were referenced to external ortho-phosphoric acid (δ 0.0) for solutions in D_2_O. COSY experiments and gradient-selected ^1^H, ^1^H total correlation spectroscopy (TOCSY, mixing time 80 ms) were recorded by use of the pulse programs cosygpqf and mlevph, respectively, with 2048 × 256 data points and 16 and 8 scans, respectively per t_1_-increment. By use of the pulse program hsqcedetgp with 2048 × 1024 data points and 16 scans per t_1_-increment multiplicity edited heteronuclear single quantum coherence spectra (HSQC) (Schleucher et al., 1994) were obtained. Heteronuclear multiple bond correlation spectra (HMBC) (Bax and Summers, 1986) were acquired using the pulse program hmbcgpndqf with 4096 × 512 data points and 64 scans per t_1_-increment and using spectral widths of 9.0 ppm for ^1^H and 222 ppm for ^13^C to check for carbonyl correlated signals.

### TagA *in Vitro* Activity Assay

To analyze the *P. alvei* TagA enzyme for its activity to transfer ManNAc from UDP-α-D-ManNAc to a α-D-GlcNAc residue as provided by the natural acceptor mimic GlcNAc-PP-(CH_2_)_11_-OPh (Xu et al., 2011), donor to acceptor ratios of 2.5:1, 1.25:1, and 1:1 were used, and MBP-TagA concentrations ranged from 50 nM to 1 μM. For this purpose, a 5-mM stock solution of UDP-α-D-ManNAc, a 2-mM stock solution of acceptor, in H_2_O, each, and a 1-μM protein solution in 25 mM bis-Tris-propane buffer, pH 7.8, were prepared. Enzymatic reactions were performed in 25 mM bis-Tris-propane buffer with addition of 250 mM NaCl (TagA reaction buffer), for 1 h at 37°C, followed by overnight-incubation at RT (Zhang et al., 2006). As controls, reaction mixtures without acceptor or enzyme were used.

For the reaction set-up, Eppendorf tubes and reagents were kept on ice throughout the pipetting procedure. A master mixture was prepared in TagA reaction buffer, containing MBP-TagA and UDP-α-D-ManNAc (0.5 mM per 40 μl of assay volume). To start the enzymatic reaction, the master mixture was added to varying volumes of the GlcNAc-PP-UndPh acceptor solution, followed by incubation as described above. The reaction was stopped by quenching with 700 μl of ice-cold H_2_O and dried *in vacuo*. Subsequently, the reactions were resuspended in 10 μl of H_2_O, each, and analyzed by thin layer chromatography (TLC), using silica G60 plates (10 cm × 5 cm; Merck) and ethyl acetate/methanol/H_2_O/acetic acid at a ratio of 4:1.5:0.7:0.1 (v/v/v/v) as solvent. The TLC plates were stained for carbohydrates with anisaldehyde-sulfuric acid dip-solution, containing ethanol/H_2_SO_4_/acetone/*p*-anisaldehyde = 100/5/3/2 (v/v/v/v) and developed at 250°C (Stahl and Kaltenbach, 1961).

For the characterization of the TagA reaction product, the assay was scaled up, using 250 nM MBP-TagA, 2.4 mM acceptor, and 0.5 mM UDP-ManNAc. After incubation and stopping the reaction as described above, the reaction mixture was purified over a (C18) Sep-Pak cartridge (see above). Dried fractions from the MeOH elution step were once dissolved in a 1:1-solution of H_2_O and acetonitrile and analyzed by LC (C4)-ESI-MS and, second, dissolved in D_2_O for final characterization by NMR spectroscopy.

### Coupled MnaA/TagA *in Vitro* Activity Assay

MnaA-His_6_ (38 μg) and MBP-TagA (20 μg) were co-incubated for 1 h at 37°C with 2.5 mM UDP-GlcNAc in sodium phosphate buffer, pH 7.5, supplemented with 11 mM MgCl_2_, in a total reaction volume of 190 μl. The reaction was stopped by addition of 700 μl of ice-cold H_2_O, purified over a (C18) Sep-Pak cartridge and analyzed by LC(C4)-ESI-MS (see above).

### Determination of a Donor Substrate for Pyruvyltransfer by CsaB

To determine, if PEP can serve as a substrate for the *P. alvei* CsaB enzyme, *in situ* NMR measurements were performed using 10 μM CsaB-His_6_, 1.5 mM UDP-α-D-ManNAc, and 1.5 mM phospho(enol)pyruvic acid monopotassium salt, in a total volume of 600 μl of deuterated sodium phosphate buffer, pD 7.9, at 27°C. ^1^H NMR and ^31^P NMR spectra were recorded *in situ* for up to 15 h (see above).

### Enzymatic One-Pot Reaction for the *in Vitro* Reconstitution of a Complete SCWP Repeat

**SCHEME 1:**
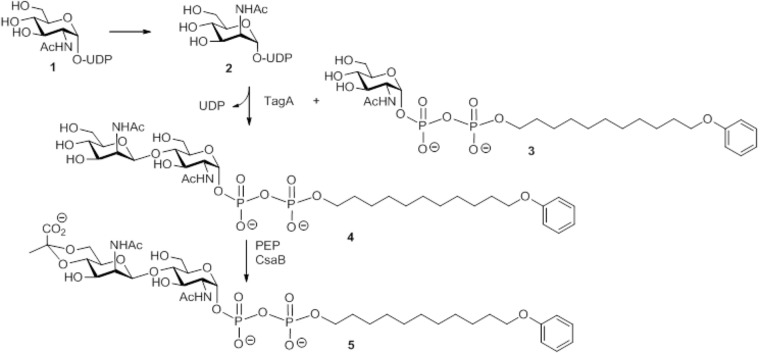
Schematic representation of the multi-enzyme assay for reconstitution of a lipid-linked SCWP repeat precursor analog using the substrates UDP-GlcNAc and PEP, the acceptor substrate GlcNAc-PP-UndPh, and the purified recombinant enzymes MnaA-His_6_, MBP-TagA, and CsaB-His_6_ from the *P. alvei* CCM 2051^T^ SCWP biosynthesis pathway. **(1)** Uridine 5′-(2-acetamido-2-deoxy-α-D-glucopyranosyl diphosphate); **(2)** uridine 5′-(2-acetamido-2-deoxy-α-D-mannopyranosyl diphosphate); **(3)** sodium 11-phenoxyundecyl (2-acetamido-2-deoxy-α-D-glucopyranosyl diphosphate); **(4)** sodium 11-phenoxyundecyl (2-acetamido-2-deoxy-β-D-mannopyranosyl-(1→4)-2-acetamido-2-deoxy-α-D-glucopyranosyl diphosphate); **(5)** sodium 11-phenoxyundecyl {2-acetamido-2-deoxy-4,6-O-[(S)-1-carboxyethylidene]-[β-D-mannopyranosyl-(1→4)]-2-acetamido-2-deoxy-α-D-glucopyranosyl diphosphate}.

To reconstitute a complete, lipid-linked repeat precursor of the *P. alvei* SCWP *in vitro* by means of the bacterium’s native enzymes, 45 μg/50 μg (the first value refers to MS analysis, the second to NMR spectroscopy) of MnaA-His_6_, 13.5 μg/45 μg of MBP-TagA and 14 μg/45 μg of CsaB-His_6_ were incubated for 1 h at 37°C together with UDP-GlcNAc (8.1 mM/8.5 mM), PEP monopotassium salt (4.2 mM/4.3 mM, Sigma-Aldrich) and GlcNAc-PP-UndPh (300 μM/1.4 mM; **Scheme 1**, compound **3**), in a total volume of 295 μl/1175 μl of 30 mM sodium phosphate buffer, pH 7.5, supplemented with 5.6 mM/5.3 mM MgCl_2_. Product formation was analyzed after (C18) Sep-Pak purification either by LC(C4)-ESI-MS or by NMR spectroscopy.

## Results

### Transcription Analysis of *csaB*, *tagA*, and *tagO* Encoded in the *P. alvei* SCWP Biosynthesis Locus

**FIGURE 1:**
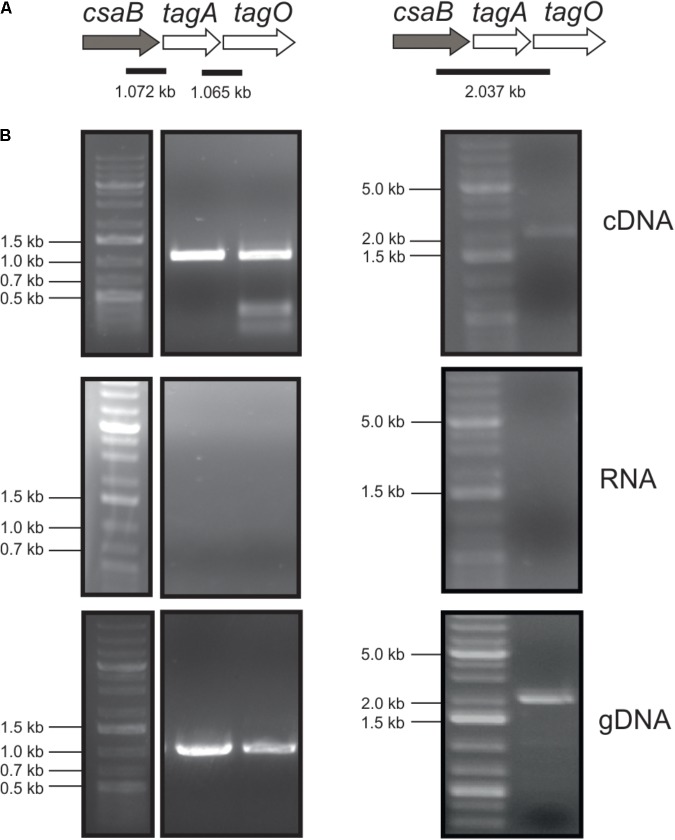
Co-transcription analysis of the *tagO*, *tagA*, and *csaB* genes from the *P. alvei* CCM 2051 SCWP biosynthesis gene locus. **(A)** SCWP gene locus with expected PCR fragment sizes indicated. **(B)** Agarose gel electrophoresis analyses of co-transcription of genes from cDNA (upper panel), total RNA (middle panel; negative control) and gDNA (lower panel; positive control). All samples were run with a standard on the same gel. Primers used are listed in **Table 1**. O’Gene Ruler 1 kb Plus DNA Ladder (Thermo Fisher Scientific) was used as a gene ladder and is indicated on the left.

To analyze whether the *csaB* (PAV_RS07425; coding for a putative pyruvyltransferase), *tagA* (PAV_RS07420; coding for a putative β-1,4 ManNAc transferase), and *tagO* (PAV_RS07415; coding for a putative initiation enzyme of SCWP biosynthesis) genes from the predicted *P. alvei* SCWP biosynthesis gene locus (Zarschler et al., 2010) are co-transcribed on a polycistronic mRNA, total RNA from *P. alvei* cells was extracted, and co-transcription of the genes was analyzed using RT-PCR as outlined in **Figure 1**. The results showed that *csaB*, *tagA*, and *tagO* are co-transcribed as a single RNA transcript (**Figure 1**), since a PCR product of the expected size was obtained with the primer pairs csaB_for/tagA_rev (1.072 kb) and tagA_for/ tagO_rev (1.065 kb), respectively, designed to bridge the ends between the ORFs of neighboring genes yielding amplification products only when co-transcription was happening. Further, primer pair csaB_for/tagO_rev yielded a 2037 bp transcript corresponding to the size of all three genes together. This confirmed that the *P. alvei tagO*, *tagA*, and *csaB* genes are transcriptionally linked.

### Overexpression and Purification of Recombinant *mnaA*, *tagA*, and *csaB*

**FIGURE 2:**
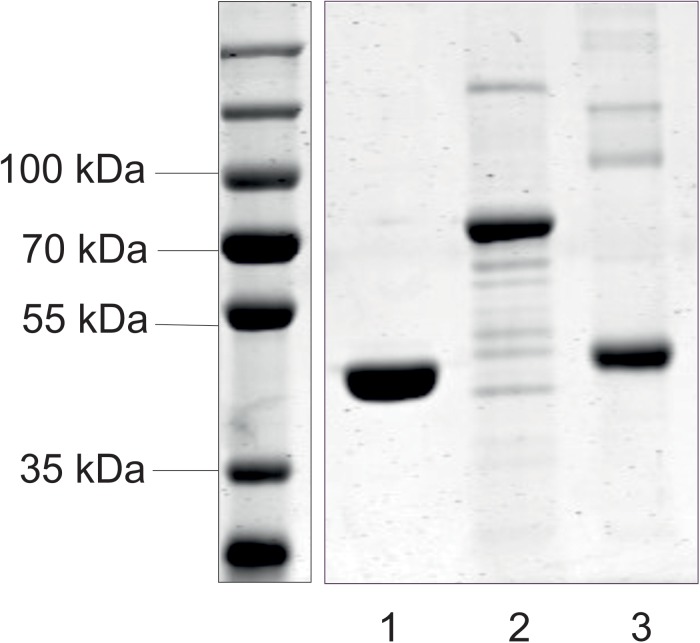
SDS-PAGE analysis of purified recombinant enzymes from the *P. alvei* CCM 2051^T^ SCWP biosynthesis pathway. MnaA-His_6_ (lane 1), MBP-TagA (lane 2), and CsaB-His_6_ (lane 3) were run on a 10% SDS-PAGE gel and visualized with Coomassie Brilliant Blue G250 staining. Standard, PageRuler Prestained Plus (left). All samples were run with a standard on the same gel.

The *mnaA* and *csaB* genes from *P. alvei* were cloned into pET22-b(+), the *tagA* gene into pMALc2e. The enzymes were produced in *E. coli* BL21(DE3) as either C-terminally His_6_-tagged proteins or, in the case of TagA, as an N-terminal MBP fusion protein, which enabled purification via nickel affinity chromatography or over an amylose resin, respectively. According to a Coomassie Brilliant Blue G250-stained 10% SDS-PAGE gel, the enzymes were purified to a high degree and revealed molecular weights of 44.5 kDa (MnaA-His_6_), 43.6 kDa (CsaB-His_6_), and 71.8 kDa (MBP-TagA), respectively (**Figure 2**), which corresponded to the values as calculated based on amino acid sequences. Proteins were verified by Western-blotting (not shown). The recombinant enzymes were stored at a concentration of 0.3 mg/ml in 20 mM sodium phosphate buffer, pH 7.5, at -20°C (MnaA-His_6_) or, in the case of enzyme inactivation upon freezing, at 4°C (MBP–TagA and CsaB-His_6_).

### Chemical Synthesis of UDP-α-D-ManNAc

For the envisaged *in vitro* reconstitution of the lipid-linked →3)-4,6-Pyr-β-D-ManNAc-(1→4)-β-D-GlcNAc-(1→ repeat of the *P. alvei* SCWP, UDP-α-D-ManNAc as the predicted donor substrate for the TagA enzyme was chemically synthesized, since it is not commercially available (Supplementary Scheme S1). Starting from 2-(acetylamido)-2-deoxy-D-mannopyranose (ManNAc) (Supplementary Scheme S1, compound **6**), UDP-α-D-ManNAc (Supplementary Scheme S1, compound **8**) was chemically synthesized in five steps and was obtained after purification via ion exchange chromatography (Ginsberg et al., 2006).

### Recombinant *P. alvei* MnaA Shows UDP-GlcNAc-2-Epimerase Activity

Purified, His_6_-tagged MnaA was mixed with the UDP-GlcNAc substrate at different concentrations in phosphate buffer, pH 7.5, supplemented with MgCl_2_, and incubated at 37°C up to 30 min; reactions were stopped in 5-min intervals.

**FIGURE 3:**
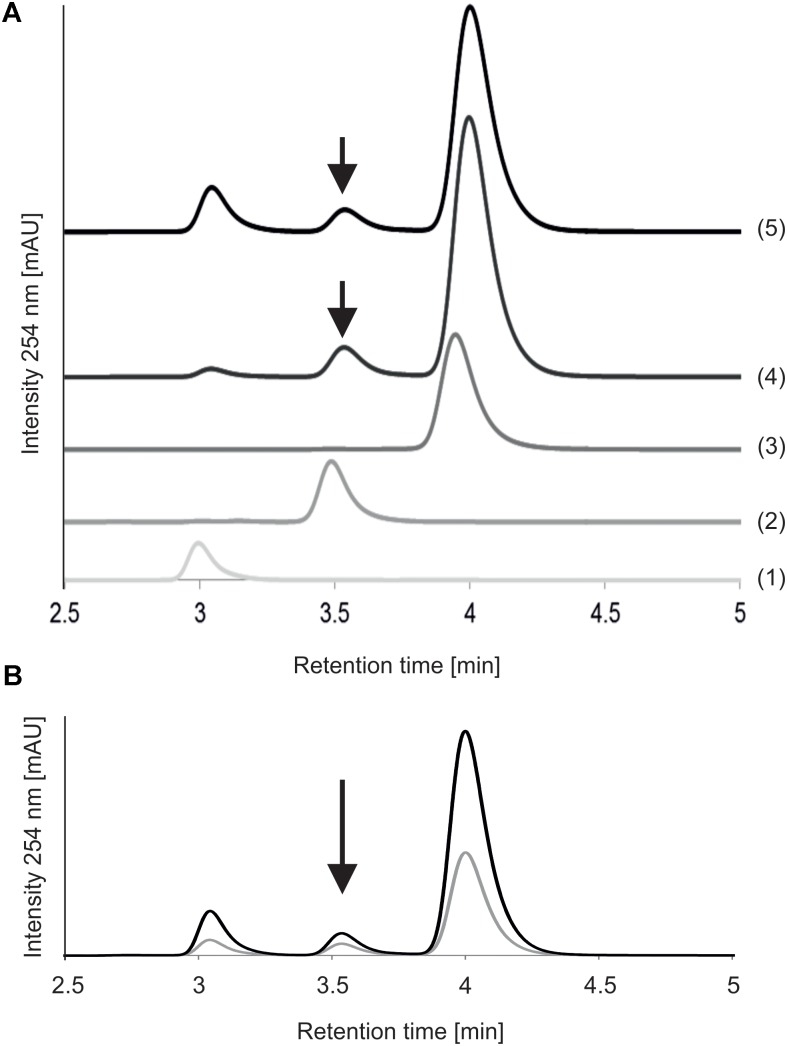
Reversed-phase HPLC analysis of MnaA-catalyzed UDP-GlcNAc epimerization. **(A)** Reactions with different reaction times. MnaA-His_6_ was incubated with 1.3 mM UDP-GlcNAc in 46 mM sodium phosphate buffer, pH 7.5, supplemented with 11 mM MgCl_2_ at 37°C. (1) UDP (standard); (2) UDP-ManNAc (standard); (3) UDP-GlcNAc (standard); (4) product formation after 5 min of reaction time; (5) product formation after 30 min of reaction time. **(B)** Reactions with different UDP-GlcNAc concentrations. MnaA-His_6_ was incubated with 0.5 mM UDP-GlcNAc (gray line) and 1.3 mM UDP-GlcNAc (black line) for 30 min as described above. Separation of the reaction mixtures from **(A,B)** was done using a Hyperclone 5 μ ODS column and products were identified based on their retention time using authentic standards. Product formation is indicated with an arrow.

MnaA-catalyzed epimerization of UDP-GlcNAc – which had a retention time of 3.9 min on the RP(C18)-HPLC column under the given conditions - was compared after 5 and 30 min of reaction time, using an authentic UDP-α-D-ManNAc standard (retention time, 3.4 min) for product identification (**Figure 3A**). Already after 5 min of incubation with the epimerase, two new peaks were detected, at 3.0 and 3.5 min, respectively, with the former corresponding to UDP and the latter to the UDP-α-D-ManNAc product. While the UDP peak increased upon prolonged reaction time, the peaks of UDP-GlcNAc and produced UDP-ManNAc decreased. Neither peak was detected when MnaA was omitted from the reaction (not shown). MnaA-catalyzed formation of UDP-α-D-ManNAc slightly increased when more substrate was provided (0.5 mM compared to 1.3 mM UDP-GlcNAc; **Figure 3B**). For further investigations, UDP-ManNAc production was established using *P. alvei* MnaA and 1.3 mM UDP-GlcNAc, revealing an epimerization rate of 9.5%, which was within the published range (Morgan et al., 1997; Murkin et al., 2004; Mann et al., 2016). Despite MnaA is annotated as non-hydrolyzing 2-epimerase, increasing amounts of UDP emerged over time as a reaction intermediate (**Figure 3A**). This was also observed by others (Morgan et al., 1997) and can be explained by the proposed mechanism B of co-factor independent epimerases involving acetamidoglucal and UDP as intermediates (Samuel and Tanner, 2002).

### Recombinant *P. alvei* TagA Shows UDP-ManNAc:GlcNAc-Pyrophosphate-R Transferase Activity

Next, the option of pyruvylation at the disaccharide stage was studied, which required the presence of a biosynthetic surrogate of the undecaprenyl diphosphate activated substrates. Previously, it had been shown that synthetic GlcNAc-PP-UndPh (**Scheme 1**, compound **3**) served as glycosyl acceptor for a UDP-Gal:GlcNAc-α-pyrophosphate-R β(1,3)-galactosyltransferase WbbD from *E. coli* strain VW187 (O7:K1) involved in the biosynthesis of O7-specific lipopolysaccharide (Riley et al., 2005) and was, thus, selected to analyze whether it would also serve as an acceptor for the UDP-ManNAc transferase TagA from *P. alvei* to produce the lipid-linked disaccharide β-D-ManNAc-(1→4)-α-GlcNAc-PP-UndPh (**Scheme 1**, compound **4**). TagA was tested for its ManNAc transfer activity and shown to be active in a concentration range of 1 μM to 50 nM. The activity was visualized by TLC showing complete conversion to product species with a donor (synthetically prepared UDP-α-ManNAc) to acceptor (**Scheme 1**, compound **3**) ratio of 2.5:1, whereas ratios of 1.25:1 and 1:1 still showed unused substrate (data not shown). LC-MS data of the (C18) SepPak-purified product mixture obtained with the 2.5:1-reaction (optimal ratio) in the negative ion mode indicated the presence of the glycosylated product (**Scheme 1**, compound **4**, *m/z* = 829.3; Supplementary Figure S1B) as well as, with suboptimal donor to acceptor ratio, unreacted substrate (compound **3** of **Scheme 1**, *m/z* = 626.6; Supplementary Figure S1A); the latter was confirmed by NMR spectroscopic data and indicated a ∼2:1 ratio for compound **3** to compound **4** (**Scheme 1**).

Furthermore, a HSQC spectrum (data not shown) identified a downfield-shifted signal at 79.0 ppm consistent with a 4-*O*-glycosylated α-GlcNAc moiety. The presence of a product mixture and of residual bis-Tris-propane buffer, however, precluded a full assignment of the ^1^H and ^13^C NMR signals, although several correlations could be established on the basis of COSY and HSQC data (**Table 2**).

**Table 2:** NMR data of compounds **4** and **5** recorded in D_2_O.

	H-1	H-2	H-3	H-4	H-5	H-6a	H-6b
	*J*	*J*	*J*	*J*	*J*	*J*	*J*
	C-1	C-2	C-3	C-4	C-5	C-6	
**Compound 4**							
β-D-ManNAc*p*-(1→	4.87	4.575	∼3.80	3.48	3.39	n.d.	n.d.
	<1.0	4.2	n.d.	9.8	2.0, 4.9		
	99.9	54.3		67.3	77.2		
CH_3_		2.03/0.04					
		22.7					
NHC = O	n.d.						
→4)-α-D-GlcNAc*p*-(1→PP	5.45	3.99	∼3.94	∼3.78	3.92	n.d.	n.d.
	3.1, 7.1	2.6	n.d.	n.d.	n.d.		
	94.9	54.2	72.15	79.0	72.2		
CH_3_		2.03 /0.04					
		22.7					
NHC = O	n.d.						
PP→OCH_2_CH_2_CH_2_-	∼3.90	1.60	n.d.				
	n.d.	6.2					
	67.6	30.7					
PhOCH_2_CH_2_CH_2_-	4.08	1.75	1.42				
	n.d.	6.6	6.6				
	69.5	28.8	25.9				
Additional signals (CH_2_)_2_	1.37–1.20						
	29.3						
Ph	–	7.01	7.36	7.02	7.36	7.01	
		7.1	7.3	7.6	7.3	7.1	
		115.7	130.6	122.0	130.6	115.7	
**Compound 5**							
4,6-Pyr-β-D-Man*p*NAc-(1→	4.90	4.51	3.93	3.58	3.39	4.01	∼3.72
	<1.0	4.3	4.5, 10.1	9.8	5.0, 9.9	4.9, 10.8	
	100.5	54.0	70.3	74.6	67.6	64.7	
CH_3_		2.05					
		22.75					
NHC = O	176.15						
Pyruvic acetal	–	–	1.44				
	176.2	102.6	25.5				
→4)-α-D-Glc*p*NAc-(1→PP	5.46	3.98	3.85	3.74	3.92	3.81	∼3.72
	br s	10.5	9.4, 9.4	9.0	n.d.	12.4	
	94.9	54.4	70.2	79.2	72.2	60.45	
CH_3_		2.05					
		22.75					
NHC = O	176.15					
PP→OCH_2_CH_2_CH_2_	3.91	1.60	1.33				
	n.d.	6.7	n.d.				
	67.6	30.5	29.3				
PhOCH_2_CH_2_CH_2_	4.08	1.74	1.43				
	6.6	6.8,	7.1				
	69.6	29.0					
Additional (CH_2_)_2_ signals	1.35–1.27						
	29.4						
Ph	–	7.02	7.37	7.04	7.37	7.02	
		8.3	8.0	7.5	8.0	8.3	
	158.9	115.7	130.5	122.2	130.5	115.7	

*Chemical shifts are given in ppm and coupling constants in Hz. n.d., not determined.*

### Recombinant CsaB Shows Interaction With PEP but No Pyruvyl Transfer to Free UDP-ManNAc

Possessing a high-energy phosphate bond and being involved in bacterial cell metabolism, PEP was chosen as the donor substrate for the pyruvyl transfer reaction. Studies of the pyruvyltransferase Pv1gp from *S. pombe* (Yoritsune et al., 2013) confirmed that PEP could be an appropriate substrate.

**FIGURE 4:**
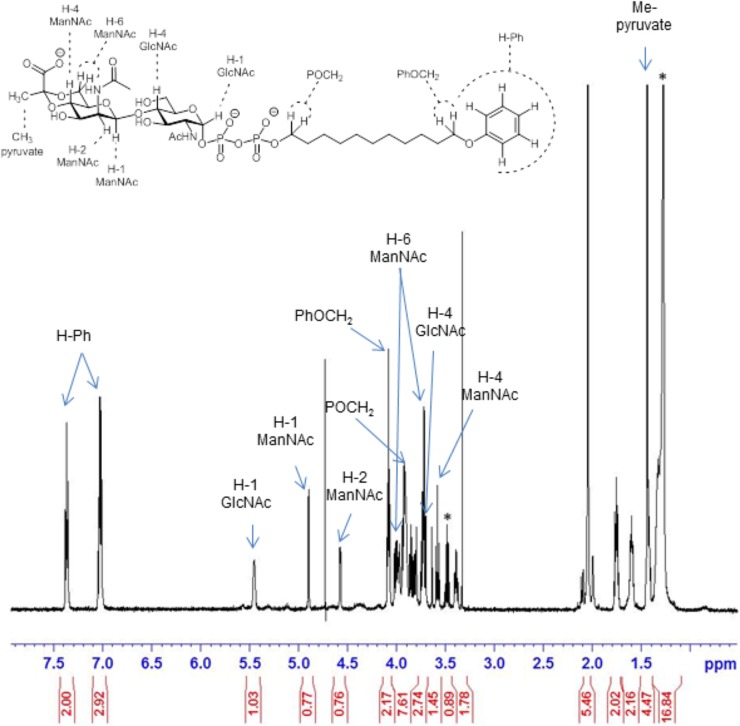
600 MHz ^1^H NMR spectrum of the pyruvylated disaccharide product 5 (from **Scheme 1**). Signals corresponding to key parts of the structure are indicated by arrows. Asterisk “^∗^” denotes residuals.

To examine, if pyruvylation occurs at the nucleotide sugar level, 1.5 mM synthetic UDP-ManNAc (Supplementary Scheme S1, compound **8**) was incubated with 10 μM CsaB and 1.5 mM PEP at pD 7.9 and 27°C in an *in situ* NMR experiment. Over the course of several hours, signals arising from UDP-ManNAc remained unchanged, but PEP was subject to a slow deuterium exchange reaction. Whereas both olefinic protons of PEP were readily seen at 5.33 and 5.15 ppm at the start of the measurement, a steady decrease of signal intensities was observed with concomitant increase of two slightly high-field shifted singlets corresponding to the monodeuterated PEP derivatives. After 14 h of reaction time, signals of protonated PEP species were almost completely absent (**Figure 4**), whereas in the control experiment without addition of enzyme, deuteration of PEP was not observed. These observations indicated an interaction of PEP with CsaB, but lack of pyruvylation of UDP-ManNAc, obviously due to the absence of an appropriate acceptor substrate.

### Choice of an Appropriate Acceptor Substrate Enables CsaB-Mediated Pyruvyl Transfer *in Vitro*

Based on the successful functional proof of the UDP-ManNAc transferase TagA by MS and NMR data, a three-step enzymatic transformation was carried out (**Scheme 1**). In a multi-enzyme assay, a one-pot reaction of the epimerase MnaA, the UDP-ManNAc transferase TagA, and the pyruvyltransferase CsaB together with UDP-GlcNAc, PEP, and the GlcNAc-PP-UndPh acceptor (**Scheme 1**, compound **3**) was set up, with the three enzymes predicted to work in a cascade reaction in nature (**Scheme 1**). The reaction product - 4,6-Pyr-β-D-ManNAc-α-D-GlcNAc-diphosphoryl-phenoxyundecyl (**Scheme 1**, compound **5**) - was purified via a (C18) Sep-Pak column, from which it eluted with water – while ManNAc-GlcNAc-PP-UndPh and remaining acceptor were eluted with methanol - and was analyzed by LC-(C4)-ESI-MS. The earlier elution of the pyruvylated product species can be explained by its higher polarity than the initial acceptor (**Scheme 1**, compound **3**) and the TagA product (**Scheme 1**, compound **4**). The mass ion *m/z* = 899.3 detected in negative ion mode in LC-(C4)-ESI-MS analysis indicated the presence of a lipid-linked pyruvylated disaccharide species (Supplementary Figure S1C).

**FIGURE 5:**
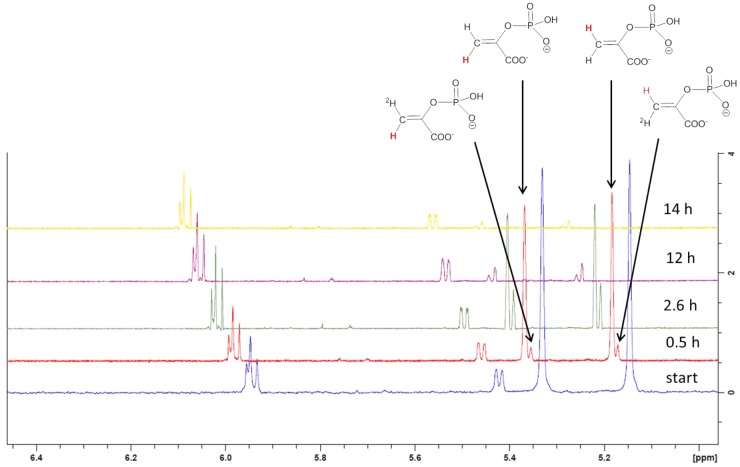
Stacked expansion plot of the 600 MHz ^1^H NMR spectrum recording the *in situ* deuterium exchange reaction of PEP in the presence of CsaB and UDP-ManNAc. Reaction times were 0.5, 2.6, 12, and 14 h. Formation of monodeuterated species is indicated by arrows in the spectrum recorded after 0.5 h reaction time as well as the assignment of the non-deuterated protons in PEP (marked in red color).

Structural analysis of the CsaB product (**Scheme 1**, compound **5**) was performed by one- and two-dimensional NMR spectroscopy of a scaled-up reaction. Briefly, the 600 MHz ^1^H NMR spectrum (**Figure 5**) recorded in D_2_O in a Shigemi tube showed *inter alia* five aromatic signals corresponding to the undecyl terminal phenyl protons, the anomeric proton of the GlcNAc unit at 5.46 ppm (as broad signal due to spin coupling with the adjacent phosphate) and the anomeric signal of the ManNAc residue at 4.90 ppm, which was correlated to H-2 of ManNAc seen at 4.51 ppm. The anomeric configurations were confirmed on the basis of the heteronuclear coupling constants *J*_C-1,H-1_ (174.2 Hz for the α-GlcNAc unit and 164.7 Hz for the β-ManNAc residue), thereby also confirming that TagA had reacted as an inverting glycosyltransferase. TOCSY correlations then allowed tracking down the spin systems originating from both anomeric protons (see **Table 2**). The high-field region showed two methyl group signals attributed to the two *N*-acetylamino groups as well as two vicinal methylene groups, each connected to an OCH_2_ signal occurring at 4.08 and 3.91 ppm, respectively. Additional CH_2_ signals were observed at 1.43 ppm and in the range of 1.34–1.27 ppm. Notably, a singlet signal corresponding to the methyl group of the pyruvyl moiety was detected at 1.44 ppm.

**FIGURE 6:**
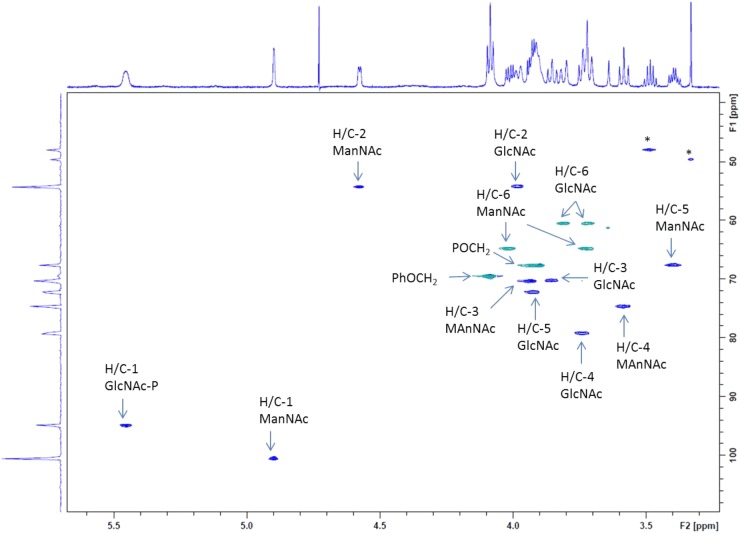
Expansion plot of the gradient-enhanced multiplicity edited HSQC spectrum of the disaccharide product **5** (from **Scheme 1**). Arabic numerals denote protons and carbons at the respective pyranose position. CH_2_-groups are presented in green color, CH-signals in blue. Asterisk “^∗^” denotes residuals.

Carbon 4 of the GlcNAc residue was observed at 79.2 ppm which, again, identified this position as the glycosylation site. In addition, the signals of carbon 6 and carbon 4 of the ManNAc residue were shifted downfield (64.7 and 74.7 ppm, respectively), which indicated that both positions had been substituted. By contrast, carbon 5 of ManNAc was significantly shifted to higher field (67.6 ppm), again in agreement with a 4,6-*O*-substitution pattern (**Figure 6**). Further structural proof was derived from HMBC measurements (data not shown), which allowed to assign the spacer-linked OCH_2_ group at 4.08 ppm as the phenoxy-linked methylene group (based on the HMBC-correlation to the quaternary aromatic carbon at 158.9 ppm), whereas the second OCH_2_ group at 3.91 ppm showed HMBC connectivity to the anomeric carbon of the GlcNAc unit at 94.9 ppm. Eventually, the pyruvic acetal group was unambiguously confirmed on the basis of HMBC-correlations of the pyruvyl methyl group to a quaternary carbon signal at 102.6 ppm and the carboxylate signal at 176.2 ppm. Moreover, an HMBC connectivity of H-6a of the ManNAc unit to C-2 of the pyruvic acid acetal was observed. The (*S*) stereochemistry of the pyruvic acid acetal was determined on the basis of the characteristic ^1^H NMR chemical shift of the methyl group at 1.44 ppm in agreement with literature data (Jansson et al., 1993).

## Discussion

Several Gram-positive bacteria, including *B. anthracis*, *B. cereus* (Forsberg et al., 2011, 2012; van Sorge et al., 2014) and the herein described non-pathogenic model organism *P. alvei* CCM 2051^T^ (Schäffer et al., 2000), attach a SCWP to their peptidoglycan cell wall. SCWPs are of strain-specific composition and typically comprise saccharide repeats (Schäffer and Messner, 2005). They are predicted to share distinct structural features with wall teichoic acids such as the murein linkage unit, and they presumably fulfill similar functions during the bacterial cell cycle (Xia et al., 2010; Brown et al., 2013). Some SCWPs are modified with pyruvate ketal groups, which endows them to serve as a cell wall ligand for SLH domains present in various Gram-positive bacterial cell surface proteins; among those and most abundant, the S-layer proteins which self-assemble into 2-dimensional crystalline arrays on the bacterial cell surface (Sleytr et al., 2010). Intriguingly, this pyruvate ketal modification is, in all investigated cases, present on a β-D-ManNAc residue (Sára, 2001). The 4,6-Pyr-β-D-ManNAc epitope might be more prevalent in SCWPs than is currently anticipated, since a frequently applied strategy for SCWP isolation is its cleavage from peptidoglycan with 48% hydrofluoric acid, which is known to liberate acid-labile pyruvate-ketal groups (Schäffer and Messner, 2017).

In *P. alvei*, the SCWP consists of pyruvylated [→3)-β-D-Man*p*NAc-(1→4)-β-D-Glc*p*NAc-(1→] disaccharide repeats (Schäffer and Messner, 2005; Janesch et al., 2013a). The SCWP repeats of *B. anthracis* (Mesnage et al., 2000; Forsberg et al., 2012) and *B. cereus* strains (Choudhury et al., 2006; Forsberg et al., 2011) as analyzed from hydrofluoric acid-treated material are extensions of the disaccharide motif by one α-D-GlcNAc residue yielding [→4)-β-D-Man*p*NAc-(1→4)-β-D-Glc*p*NAc-(1→6)-α-D-Glc*p*NAc-(1→] and include additional non-stoichiometric galactosyl (Chateau et al., 2018) and acetyl modifications; in these SCWPs, exclusively the non-reducing-end β-D-ManNAc residue carries a 4,6-linked pyruvate ketal modification.

Given that SCWPs comprise a large fraction of the Gram-positive cell wall - where approximately every fourth *N*-acetylmuramic acid residue of the peptidoglycan backbone is modified with an SCWP (Schäffer et al., 2000), understanding how these polymers are made and, especially, how the pyruvate ketal modification is elaborated, are necessary steps in exploring their potential as antimicrobial targets. For studying SCWP biosynthesis, *P. alvei* is an ideal model organism, since we have exact knowledge of its SCWP structure (Schäffer et al., 2000) and prediction of a genomic SCWP biosynthesis gene locus (Zarschler et al., 2010), which makes a chemo-enzymatic approach feasible.

Pyruvylated SCWPs fall into the category of anionic cell wall glycopolymers - to which also wall teichoic and lipoteichoic acids belong - the presence of which seems to be essential for the Gram-positive cell wall (Chapot-Chartier and Kulakauskas, 2014). This is substantiated by the failure to create a viable knock-out mutant of the *tagO* gene predictably encoding the initiation enzyme of SCWP biosynthesis, in both *B. anthracis* (Lunderberg et al., 2015; Oh et al., 2017) and *P. alvei* (Fiona F. Hager and Christina Schäffer, unpublished data); in both of these bacteria, the SCWP is the only known anionic cell wall glycopolymer. In contrast, for *B. subtilis*, a viable *tagO* knock-out mutant affecting wall teichoic acid biosynthesis could be obtained; albeit, this mutant experienced morphological changes and was unable to colonize host tissue (D’Elia et al., 2006a). Remarkably, the creation of a *B. subtilis* mutant with simultaneous deficiency in wall teichoic acid and lipoteichoic acid was lethal, probably due to complete charge depletion of the cell wall (D’Elia et al., 2006a).

We have obtained evidence that the principle of TagO and TagA catalyzed formation of a undecaprenylpyrophosphate-linked ManNAc-GlcNAc disaccharide as known from teichoic acid biosynthesis (Brown et al., 2013) is also valid for SCWP biosynthesis, supporting bioinformatic predictions (Zarschler et al., 2010; Oh et al., 2017). In the current study, this picture is even extended by shedding light on the pyruvylation step, using the *P. alvei* SCWP biosynthesis enzymes TagA and CsaB in conjunction with MnaA and a GlcNAc-PP-UndPh precursor. We succeeded in producing in a multi-enzyme *in vitro* assay a complete, pyruvylated, lipid-linked SCWP repeat precursor derivative, thereby obtaining insight into the biochemical basis of CsaB activity. Earlier we proposed that the *P. alvei* genome harbors a dedicated SCWP biosynthesis gene locus. Here, we show that *csaB* (PAV_RS07425), *tagA* (PAV_RS07420), and *tagO* (PAV_RS07415) are indeed linked as one transcriptional unit (**Figure 1B**), which makes their concerted action a likely scenario; the *mnaA* gene (PAV_RS07610) is located elsewhere on the genome. The UDP-GlcNAc-2-epimerase MnaA was included in our biosynthetic *in vitro* study, since *in vivo*, UDP-GlcNAc epimerization is a necessary prerequisite for provision of the UDP-ManNAc donor substrate to TagA. For each of these genes, a single copy was identified in the *P. alvei* genome.

Despite its indispensability for assembling the cell wall of many Gram-positive bacteria, the pyruvylation reaction to obtain the 4,6-Pyr-β-D-ManNAc epitope has not been biochemically investigated, thus far. This might be due to the lack of available substrates for *in vitro* studies of the predicted pyruvyltransferase CsaB, and missing knowledge of the biosynthetic stage of pyruvylation. In an initial *in situ* NMR measurement of recombinant *P. alvei* CsaB together with PEP, interaction of the enzyme and PEP could be observed (**Figure 4**), confirming PEP as donor substrate for pyruvylation. Searching for an appropriate acceptor candidate, notably, pyruvyltransfer could not be detected with synthetic pNP-β-ManNAc (kindly provided by Stephen Withers; Fiona F. Hager, unpublished data) mimicking the disaccharide linkage, nor with synthetic UDP-ManNAc, although a nucleotide binding site is predicted for CsaB based on its amino acid sequence (Fiona F. Hager, Arturo López-Guzmán, Christina Schäffer, unpublished data). These findings let us conclude that either a longer SCWP building block, or a lipid-linked precursor was needed. Since more likely from a biosynthetic perspective and supported by the current model of SCWP biosynthesis in *B. anthracis*, where non-stoichiometric modifications of the repeats would occur at the lipid-linked stage (Missiakas and Schneewind, 2017), we decided for the latter option and set up a stepwise enzymatic cascade to reach our target molecule.

An *in vitro* enzymatic assay using recombinant UDP-ManNAc:GlcNAc-lipid transferase TagA in combination with a synthetic 11-phenoxyundecyl-diphosphoryl-α-GlcNAc (GlcNAc-PP-UndPh, **Scheme 1**, compound **3**) acceptor (with the undecyl moiety mimicking the natural undecaprenyl carrier lipid) and chemically synthesized UDP-ManNAc or UDP-ManNAc produced by MnaA catalysis as donor resulting in a ManNAc-GlcNAc-PP-UndPh product species (**Scheme 1**, compound **4**), was established. Importantly, in a co-incubation assay of MnaA and TagA, apparently enough UDP-GlcNAc was epimerized for subsequent Tag A-catalyzed ManNAc transfer to produce compound **4**. While TagA is a predicted cytosolic protein, fluorescence microscopy studies with a TagA-GFP chimera showed its localization near the cytoplasmic membrane supporting the necessity of a lipid tail on the acceptor substrate; further, the TagA-GFP chimera was shown to accumulate at cellular septation sites underlining its activity in cell wall metabolism (Fiona F. Hager, Christina Schäffer unpublished data). It is very likely that in the native host, the TagA reaction is preceded by a TagO reaction initiating SCWP biosynthesis by transferring GlcNAc-phosphate to an undecaprenylphosphate carrier lipid (Weidenmaier et al., 2004; D’Elia et al., 2006b).

With compound **4** (**Scheme 1**) in hands we had a suitable acceptor substrate for the pyruvyl transfer reaction to generate the pyruvylated, lipid-linked disaccharide repeat precursor constituting the *P. alvei* SCWP. The product was fully elucidated by NMR analysis providing the first functional proof of a bacterial pyruvyltransferase *in vitro*. Altogether, our data suggests a mechanism where pyruvylation occurs most likely at the stage of the lipid-linked disaccharide. Currently, it is still unclear, if polymerization of the complete SCWP chain occurs in the cytoplasm prior to export followed by, predictably, LytR-CpsA-Psr family ligase-mediated (Kawai et al., 2011; Zilla et al., 2015; Schaefer et al., 2017) linkage to the C6-hydroxyl of MurNAc in the glycan strands of peptidoglycan. While during biosynthesis of wall teichoic biosynthesis, polymerization occurs at a single lipid-carrier (Xia and Peschel, 2008; Kawai et al., 2011; Chan et al., 2013), this scenario is questionable in the case of SCWP biosynthesis of *P. alvei*, where we have shown *in vitro* that the disaccharide lipid-carrier serves as a substrate for CsaB (this study). Alternatives, as known from LPS biosynthesis pathways (Raetz and Whitfield, 2002) would be the assembly of individually synthesized pyruvylated repeats and step-wise transfer of repeats to the non-reducing end of a lipid-linked primer or, polymerization after export. The requirement of a lipid carrier for pyruvyltransferase activity is supported by studies of the pyruvyltransferase WcfO from the capsular polysaccharide A biosynthesis gene cluster of *B. fragilis.* There, according to MS evidence, the enzyme is active on an undecaprenyl-pyrophosphate-linked disaccharide for producing a PvGal residue prior to final assembly of the capsular polysaccharide A tetrasaccharide repeat precursor (Sharma et al., 2017).

Another scenario is known from polyribitol wall teichoic acid biosynthesis in *S. aureus* and *B. subtilis*. It involves a primase, TagB, which attaches a single glycerol-phosphate (GroP) unit to the non-reducing end of the lipid-linked GlcNAc-ManNAc disaccharide platform (Brown et al., 2008). Following assembly of the disaccharide linkage unit, the pathway for polyribitol wall teichoic acid requires the enzymes TarF, TarK, and TarL to complete the polymeric main chain (Lazarevic et al., 2002). Once polyribitol wall teichoic acid has been completed, still attached to the undecaprenyl carrier lipid, it is flipped to the external surface of the cytoplasmic membrane (Swoboda et al., 2010) where it is linked to peptidoglycan involving a LytR-CpsA-Psr (LCP) family ligase (Kawai et al., 2011; Zilla et al., 2015; Schaefer et al., 2017).

Generally, polymerization of SCWP repeats and SCWP ligation to peptidoglycan are remaining challenging open questions in SCWP biosynthesis pathways. According to a recent model of *B. anthracis* SCWP biosynthesis (Missiakas and Schneewind, 2017; Oh et al., 2017), an undecaprenyl-pyrophosphate linked GlcNAc-GlcNAc-ManNAc trisaccharide repeat would be preassembled in a cytoplasmic, TagO and TagA1/-2 catalyzed reaction followed by membrane flipping involving the multidrug and toxin extrusion-like protein Bas5279. At the outer surface of the cytoplasmic membrane, the lipid-bound SCWP would be ligated onto murein linkage units and the final polymer would be attached to peptidoglycan by a LytR-CpsA-Psr ligase. The newly identified WpaA and WpaB proteins are anticipated to be involved in both polymerization and ligation reactions, however, without experimental evidence of activity. These proteins are categorized as Pfam protein family PF13425, which belongs to clan CL0499 that also encompasses Wzy (polymerases) and WaaL (ligases) proteins involved in bacterial polysaccharide biosynthesis, such as LPS (Raetz and Whitfield, 2002). Depletion of either protein was shown to affect vegetative growth, cell shape, and S-layer assembly, supportive of a role of these proteins in *B. anthracis* cell wall metabolism (Oh et al., 2017). Strikingly, the *B. anthracis* SCWP biosynthesis model does not take into account the pyruvylation reaction.

We provided the first functional proof of a bacterial pyruvyltransferase CsaB by a stepwise enzymatic synthesis of the required acceptor substrate, taking the structurally defined SCWP of *P. alvei* as a model system. In a one pot reaction applying a reaction cascade of UDP-GlcNAc epimerization and ManNAc transfer, a lipid-pyrophosphate linked ManNAc-GlcNAc disaccharide species was generated whereon CsaB-catalyzed pyruvyl transfer was executed. This is a key step in deciphering the biosynthesis of pyruvylated SCWPs as found not only in our model organism but also in Gram-positive pathogens, where due to the essentiality of the SCWP, the mechanistic characterization of the involved enzymes may reveal possible antimicrobial targets.

## Author Contributions

FH, PK, and CS conceived and designed the experiments. FH, MP, SK, and AL-G performed the experiments and developed methodology. FH, SK, MB, MP, PK, and CS analyzed the data. SK and IB contributed reagents, materials, and analysis tools. FH, PK, and CS wrote the manuscript. All authors revised and approved the manuscript.

## Conflict of Interest Statement

The authors declare that the research was conducted in the absence of any commercial or financial relationships that could be construed as a potential conflict of interest.

## Footnotes

1 http://web.expasy.org/protparam

## Abbreviations

cDNA: copy DNA
COSY: correlation spectroscopy
gDNA: genomic DNA
GlcNAc-PP-UndPh: 11-phenoxyundecyl-diphosphoryl-α-GlcNAc
His_6_-tag: hexahistine tag
HMBC: heteronuclear multiple-bond correlation spectroscopy
HSQC: heteronuclear single-quantum coherence spectroscopy
IPTG: isopropyl-β-D-thiogalactopyranoside
ORF: open reading frame
PEP: phosphoenolpyruvate
PvGal: 4,6-ketal-linked galactose
RT: room temperature (22°C)
SCWP: secondary cell wall polymer
SDS-PAGE: sodium dodecyl sulfate-polyacrylamide gel electrophoresis
S-layer: bacterial cell surface layer
SLH domain: S-layer homology domain
TOCSY: total correlation spectroscopy
