# Supplementary material for: Functional Characterization of Enzymatic Steps Involved in Pyruvylation of Bacterial Secondary Cell Wall Polymer Fragments
Source: Front Microbiol. 2018 Jun 27;9:1356. doi: 10.3389/fmicb.2018.01356 (PMC6030368; doi:10.3389/fmicb.2018.01356)
Supplement: Supplementary file 1 [file Data_Sheet_1.PDF]

Supplementary Material

Functional Characterization of Enzymatic Steps Involved in Pyruvylation of Bacterial Secondary Cell Wall Polymer Fragments

Fiona F. Hager[1], Arturo López-Guzmán[1], Simon Krauter[2], Markus Blaukopf[2], Mathias Polter[1], Inka Brockhausen[3], Paul Kosma[2], and Christina Schäffer[1]*

[1] *NanoGlycobiology* unit, Department of NanoBiotechnology, Universität für Bodenkultur Wien, Muthgasse 11, A-1190 Vienna, Austria

[2] Department of Chemistry, Institute of Organic Chemistry, Universität für Bodenkultur Wien, Muthgasse 18, A-1190 Vienna, Austria

[3] Department of Biomedical and Molecular Sciences, Queen's University, Kingston K7L3N6, ON, Canada

* **Correspondence:** Christina Schäffer, christina.schaeffer@boku.ac.at

Supplementary Data

Supplementary Scheme S1
Supplementary Figure S1

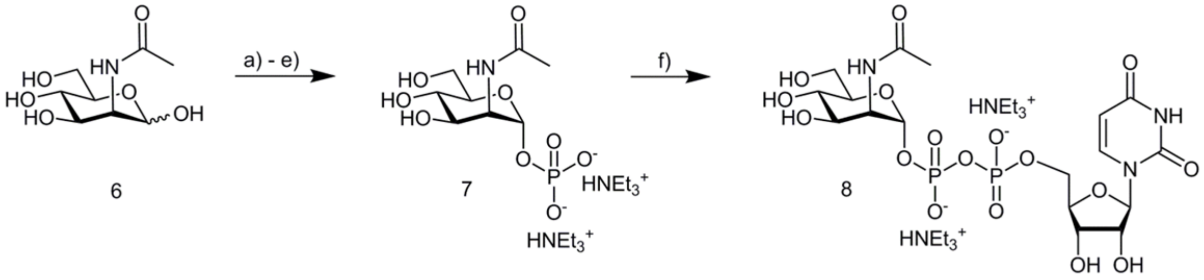

Supplementary SCHEME S1 │ Schematic representation of UDP-α-D-ManNAc synthesis. (a) Acetic anhydride, pyridine, 4-(dimethylamino)-pyridine, 21.5 h, room temperature, 85%; (b) trifluoromethanesulfonic acid, dichloromethane, 2 h, 75%; (c) benzyl phosphate, toluene, 6 d, room temperature, 28%; (d) H_2, Pd on active charcoal, methanol, room temperature, quantitative; (e) 0.25 M triethylammonium bicarbonate buffer, pH 8.0; (f) UMP morpholidate, pyridine, 9 d, room temperature, 19%. (6) 2-Acetamido-2-deoxy-D-mannopyranose; (7) Triethylammonium 2-acetamido-2-deoxy-α-D-mannopyranosyl phosphate; (8) Triethylammonium uridine 5'-(2-acetamido-2-deoxy-α-D-mannopyranosyl diphosphate).

A

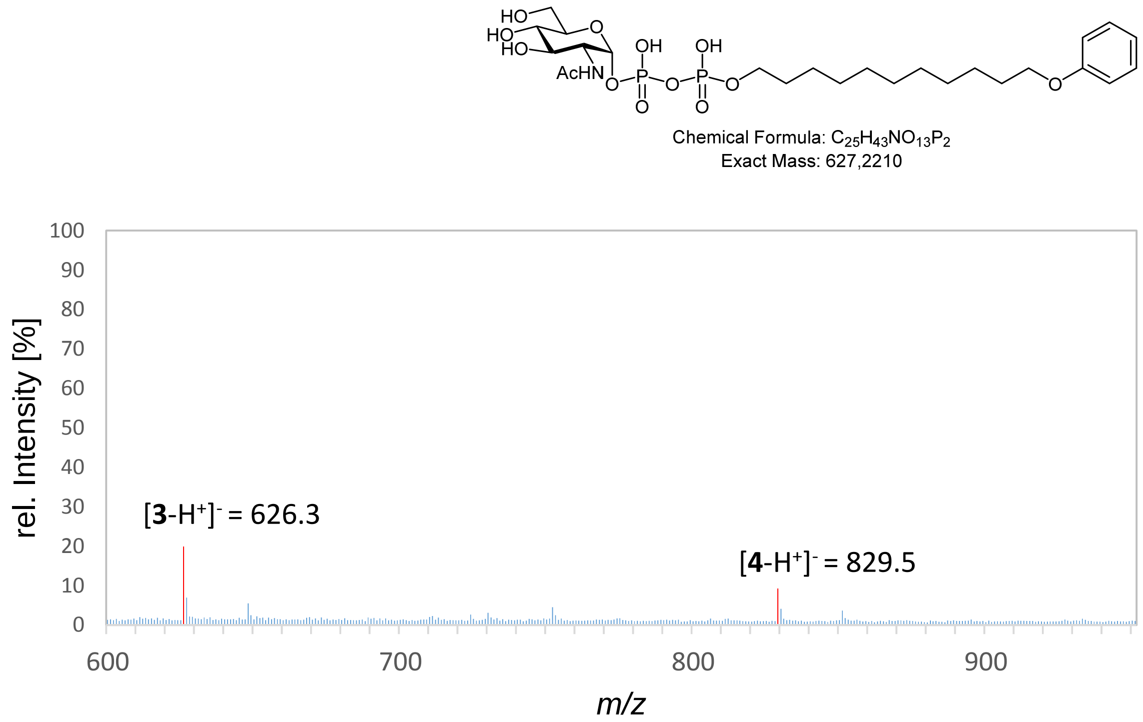

Chemical Formula: C25H43NO13P2
Exact Mass: 627,2210
rel. Intensity [%]
[3-H+]- = 626.3
[4-H+]- = 829.5
m/z

B

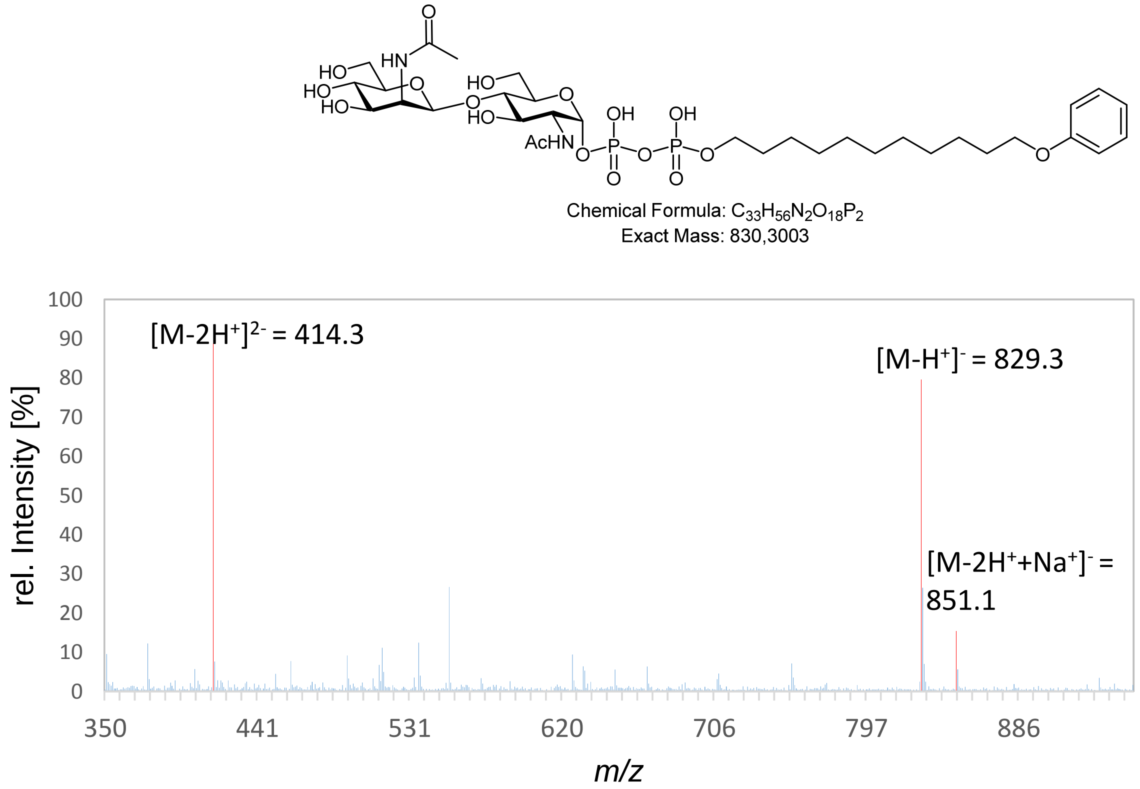

Chemical Formula: C33H56N2O18P2
Exact Mass: 830,3003
rel. Intensity [%]
[M-2H+]2- = 414.3
[M-H+]- = 829.3
[M-2H++Na+]- = 851.1
m/z

C

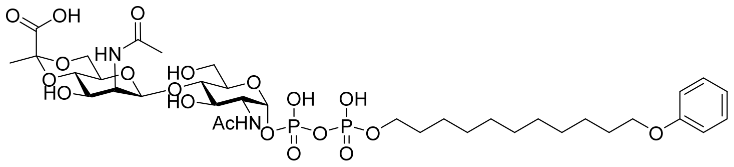

Chemical Formula: $C_{36}H_{58}N_2O_{20}P_2$
Exact Mass: 900,3058

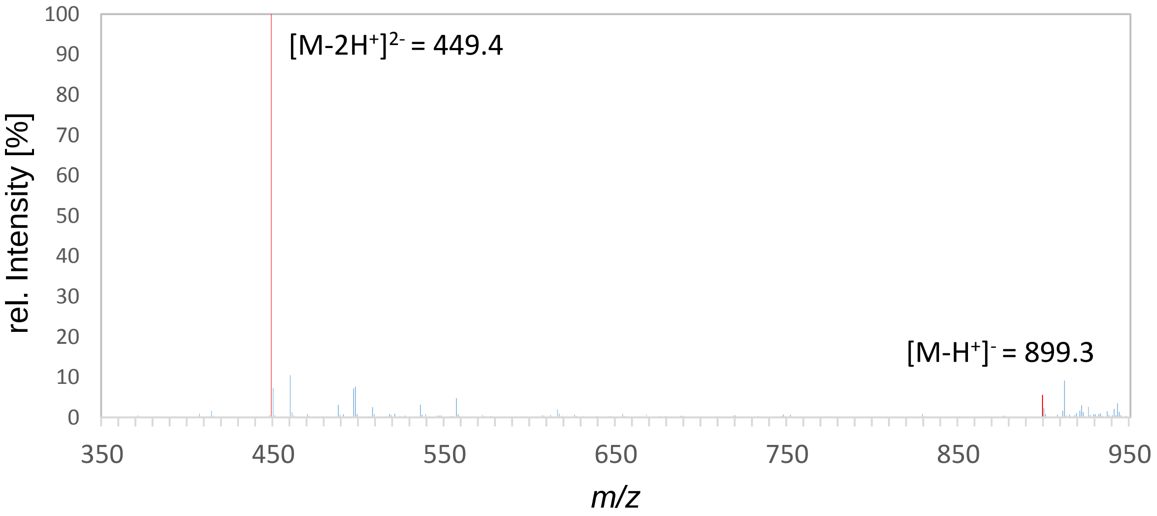

Supplementary FIGURE S1 │ (A) MS spectrum of unreacted TagA acceptor substrate (compound **3** from Scheme 1, $[M\text{-}H^+]^-$ = 626.3). **(B)** MS spectrum of TagA product (compound **4** from Scheme 1, $[M\text{-}H^+]^-$ = 829.3 $[M\text{-}2H^++Na^+]^-$ = 851.4 and $[M\text{-}2H^+]^{2-}$ = 414.3). **(C)** MS spectrum of pyruvylated lipid-linked disaccharide (compound **5** from Scheme 1, $[M\text{-}H^+]^-$ = 899.3 and $[M\text{-}2H^+]^{2-}$ = 449.4).
